# The Elements of Anatomy

**Published:** 1835-01-01

**Authors:** 


*The Elements of Anatomy.* By Jones Quain, m.d., Professor of Anatomy and Physiology in the University of London. *Third Edition, revised and enlarged.*—London, 1834. 8vo. pp. 870.

In reviewing a work like the one before us, our task is an easy and an agreeable one: we have merely to announce to our readers that an excellent book has again received the benefit of the author's revision, and to recommend those who are in want of a compendium of anatomy to procure Dr. Quain's Elements. Our only difficulty consists in selecting a passage for extract from a book of this kind; and we shall therefore quote our author's account of Mr. Kiernan's discoveries, although we gave them at some length in a former Number. Our readers will probably excuse this; for, as minute structure is not very easily comprehended from mere description, those who have not had the opportunity of seeing those hepatic ramifications demonstrated under the microscope, will be glad to have them presented again in an abridged form.

"The recent researches of Mr. Kiernan give a very clear view of several points connected with the distribution of the vessels in the liver, and particularly of the structure of the lobuli. It is obvious that the interior of the organ is channelled or hollowed into two sets of canals; the one giving lodgment to the hepatic veins, the other to the portal vein, its branches, and the accompanying arteries and ducts. At the transverse fissure, the vein, duct, and artery divide into branches which enter the portal canals. These divide and subdivide into smaller branches, which enter the smaller canals; and every canal, however small, contains an offset from the portal vein, the hepatic artery, and the duct. These are enclosed by Glisson's capsule, which lines the portal canals, and forms sheaths for the larger vessels, and a web in which the smaller vessels ramify; it enters the interlobular fissures, forming capsules for the lobules, and finally extends into their interior, and, with the blood-vessels, expands itself over the secreting biliary ducts. 'The capsule thus presents three portions,—a vaginal, interlobular, and lobular portion; and, as the vessels ramify in the capsule, their branches admit of a similar division.'

"The hepatic ducts can be traced along the canals, in the fissures between the lobules, and into the lobules, where they form plexuses. 'These may be called the lobular biliary or secreting plexuses, as being the immediate agents in the secretion of bile.' The branches of the portal vein, and the hepatic arteries, also enter the lobules. The venous branches form a plexus, which communicates with the incipient radicles of the hepatic vein; and the arteries, which are very few and very minute, are the nutrient vessels of the lobules, and probably terminate in the plexus of the portal vein. The branches of the artery ramify freely upon the coats of the portal vein, and on the hepatic ducts, furnishing materials for the nutrition of both, and to the latter for the secretion of mucus which lubricates their interior.

"The trunks of the hepatic veins are lodged in the 'hepatic-venous canals.' Their incipient radicles commence in the interior of the lobules, so that each lobule may be said to be 'sessile' upon a minute venous branch. Hence, when a hepatic vein is laid open, the orifice of each minute branch which terminates in it is seen to come out of the middle of a lobule; but the branches of the portal vein, when viewed in the same way, correspond with the interstices between the lobules.

"*Structure of the Lobules*. Each lobule is found to consist of a reticulated plexus, formed by the minute radicles of the biliary ducts. For these, when examined with a high magnifying power, are seen to divide and subdivide, so as to form a mesh in its interior, which is supported by a nidus of cellular tissue furnished by Glisson's capsule. Upon this mesh or plexus is disposed another, formed by the terminal branches of the portal vein. This is the 'lobular venous plexus,' from which that formed by the ducts can be distinguished, as the latter presents somewhat the appearance of cells. The branches of the venous plexus converge from the circumference of the lobule towards its centre, and communicate with the incipient radicles of the hepatic vein.

"It is difficult to inject the ducts, owing to their being filled with bile. Mr. Kiernan succeeds by first tying the portal vein and hepatic artery in a living animal, after feeding it. By this expedient the secretion of bile is suspended, and that which the ducts contain is discharged. The ducts cannot be injected directly from the hepatic vein, for no branches of this vessel ramify on their coats. Whenever it does reach the ducts, it is only through the branches of the portal vein which spread upon them; and, even when the ducts are injected from the portal vein or the hepatic artery, the fluid gets into their interior, by rupturing their lining membrane.

"The residue of the blood conveyed by the hepatic artery to the lobules, to the different vessels, and the ducts, for their nutrition, is taken up by minute veins, and conveyed into the portal vein, so that part of the blood from which bile is secreted is derived from the liver itself." (P. 650.)

We quote the following passage, as it gives us an opportunity of correcting some common but mistaken opinions as to the doctrines of the ancients.

"*Artery, Arterial Tissue, Arteria.*—The term 'artery,' in its original acceptation, meant a tube containing air, (αηρ, air; τηρεω, to contain,) or some subtile agent, whether called archæus or vital spirits. It was at one time supposed that veins alone carried blood, as they were observed at all times to contain it, in the dead as well as the living; but as arteries were always found empty after death, they were imagined to be the conductors during life of something more refined and ethereal. Though this hypothesis has been long forgotten, the term to which it gave rise is retained even now, notwithstanding that centuries have elapsed since a very different doctrine has been completely established." (P. 69.)

We say nothing of the derivation of αρτηρια from αηρ and τηρεω, though it is an erroneous one, and belongs to an erroneous class, as αρτηρια is certainly derived, with its kindred word αορτη, from αειρω, *to lift.* The error we would wish to correct consists in supposing that the ancients were ignorant that the arteries contained blood; yet what says Aretæus, in his chapter Περι ἁιματος αναγωγης, *On the bringing up of blood?*

is a difference in the quantity thrown up, and it differs also according as it may come from an artery or a vein. For the blood is black and thick, and easily coagulable, if it proceeds from a vein, and is less dangerous, and more quickly restrained. But, if it comes from an artery, it is light-coloured and thin, and not so very coagulable, and the danger is more urgent, and the flow is not so easily stopped; for the pulses of the artery excite the hemorrhage, and the wound does not unite, on account of the frequency of the motion," &c.

Nor is this the only passage of the kind: towards the end of the chapter, Aretæus again mentions the darker colour and greater coagulability of venous blood; and, in his chapter on the cure of headach, he recommends bleeding from the arteries behind the ear, which may be distinguished, he says, by their pulsation. (*De Morb. Diut. cur.*, lib. i. cap. 2, p. 115, ed. Boerh.)

Those of our readers who may choose to embellish their leisure hours, by studying the writings of the classic physicians, will be astonished to find how groundless are the assertions respecting them which are to be found in modern works. The reason may probably be, that the original is rarely consulted, and consequently the scraps of ancient lore copied from book to book, without examination, are encumbered with additional mistakes at each transplantation, till they lose all resemblance to the archetype: like a drop of some pungent juice ten times poured from one homœopathic phial into another, and diluted each time with a hundred waters!

We again recommend Dr. Quain's Manual for general use, and are of opinion that it is one of the few books which really deserve a place in the library of every medical student.

